# Perfusion cardiovascular magnetic resonance: Comparison of an advanced, high-resolution and a standard sequence

**DOI:** 10.1186/1532-429X-14-34

**Published:** 2012-06-09

**Authors:** Geraint Morton, Masaki Ishida, Andreas Schuster, Shazia Hussain, Tobias Schaeffter, Amedeo Chiribiri, Eike Nagel

**Affiliations:** 1 King’s College London British Heart Foundation (BHF) Centre of Excellence; National Institute of Health Research (NIHR) Biomedical Research Centre at Guy’s and St. Thomas’ NHS Foundation Trust; Wellcome Trust and Engineering and Physical Sciences Research Council (EPSRC) Medical Engineering Centre; Division of Imaging Sciences and Biomedical Engineering; The Rayne Institute, St. Thomas’ Hospital, London, United Kingdom

## Abstract

**Background:**

Technical advances in perfusion cardiovascular magnetic resonance (CMR), particularly accelerated data acquisition methods, allow myocardial perfusion imaging with unprecedented spatial resolution. However, it is not clear how implementation of these recent advances affects perfusion image quality, signal and contrast to noise ratios (SNR & CNR) and the occurrence of important artefacts in routine clinical imaging. The objective of this study was therefore to compare a standard and an advanced, high-resolution perfusion sequence.

**Methods:**

A standard ultrafast gradient echo perfusion sequence (st-GrE) was compared with an advanced kt-accelerated steady state free precession sequence (kt_BLAST_-SSFP) at 1.5 T in healthy volunteers (n = 16) and in patients (n = 32) with known or suspected coronary artery disease. Volunteers were imaged with both sequences at rest and patients underwent stress and rest imaging with either st-GrE or kt_BLAST_-SSFP prior to X-ray coronary angiography.

A blinded expert scored image quality and respiratory artefact severity and also classified patients for the presence of CAD. The extent, transmurality and duration of dark rim artefacts (DRA) as well as signal to noise (SNR) and contrast to noise (CNR) were quantified.

**Results:**

In normal hearts kt_BLAST_-SSFP imaging resulted in significantly improved image quality (p = 0.003), SNR (21.0 ± 6.7 vs. 18.8 ± 6.6; p = 0.009), CNR (15.4 ± 6.1 vs. 14.0 ± 6.0; p = 0.034) and a reduced extent (p = <0.0001) and transmurality (p = 0.0001) of DRA. In patients kt_BLAST_-SSFP imaging resulted in significantly improved image quality (p = 0.012), and a reduced extent (p = <0.0001), duration (p = 0.004) and transmurality (p = <0.0001) of DRA. Sensitivity and specificity for the detection of CAD against X-ray angiography was comparable with both sequences. There was a non-significant trend towards increased respiratory artefacts with kt_BLAST_-SSFP in both patients and volunteers.

**Conclusions:**

Advanced high resolution perfusion CMR using a *k-t*-accelerated SSFP technique results in significantly improved image quality, SNR and CNR and a reduction in the extent and transmurality of DRA compared to a standard sequence. These findings support the use of advanced perfusion sequences for clinical perfusion imaging however further studies exploring whether this results in improved diagnostic accuracy are required.

## Background

Detection of myocardial ischaemia is important particularly in coronary artery disease (CAD) for diagnostic purposes, identification of patients with an adverse prognosis [1] and for guiding revascularization [2,3]. Perfusion cardiovascular magnetic resonance (CMR) has become established as a valuable, non-invasive tool for ischaemia detection that is free of ionizing radiation and is at least as reliable other imaging techniques [4-6]. However, despite this, the requirement to acquire large amounts of data in a short duration means that perfusion imaging is technically challenging, and still has important limitations. These limitations are most notably that the requirement to maintain high temporal resolution constrains spatial resolution and there is an increased tendency for imaging to be affected by problematic artefacts. Artefacts occur both as a result of the competing constraints of spatial and temporal resolution and of the method of first pass imaging itself. Imaging during the arrival of a concentrated bolus of contrast agent (CA) into the left ventricle contributes to the occurrence of dark rim artefacts (DRA). DRA are a particular concern as they can mimic or hide subendocardial defects resulting in diagnostic errors. These perfusion imaging related demands are in addition to the usual requirements for patient breath-holding, and for precise gating with the cardiac cycle, both of which can be more difficult during vasodilator stress. Respiratory artefacts as a result of inadequate breath holding can also compromise study interpretation.

Currently a number of types of sequence, each with different advantages and disadvantages, are used for perfusion imaging. Common sequences include ultrafast gradient echo sequences, single-shot echo planar imaging (EPI), hybrid EPI and more recently steady-state free precession (SSFP) sequences. Whilst some previous studies have compared perfusion sequences [7-11] CMR hardware and software has continued to evolve rapidly and it is not known whether the implementation of more recent advances can address the current limitations associated with perfusion imaging.

Parallel imaging techniques such as sensitivity encoding (SENSE) are now routinely used to accelerate perfusion imaging and this acceleration can be used to improve spatial resolution. However, in practice, this is limited to 2-fold acceleration due to associated artefact and noise penalties [12]. Advanced *k-t* acceleration techniques allow higher degrees of acceleration than SENSE and have been proposed more recently as a useful technique to improve the spatial resolution of perfusion imaging even further whilst preserving temporal resolution and cardiac coverage [13].

The objective of this study was to compare a standard perfusion sequence with an advanced, high-resolution method to determine whether there is a measurable difference in performance. We therefore compared a standard turbo field echo (st-GrE) perfusion sequence (ultrafast gradient echo sequence), to an advanced, high-resolution *k-t* BLAST accelerated balanced turbo field echo sequence (kt_BLAST_-SSFP), in normal hearts and in patients with known or suspected CAD.

## Methods

### Study population

The st-GrE and kt_BLAST_-SSFP perfusion sequences were compared in 16 volunteers and in 32 patients with known or suspected CAD. Volunteers underwent rest perfusion imaging using both the st-GRE and the kt_BLAST_-SSFP sequences and patients underwent stress and rest perfusion imaging with one of the sequences as detailed below. The local ethics committee approved the human studies and all participants gave written informed consent.

### Volunteers

Volunteers referred for a clinically indicated non-perfusion CMR scan with a high pre-test probability of a normal scan were recruited prospectively. Exclusion criteria were a contraindication to MRI (incompatible implants, weight > 150 kg, claustrophobia, inability to lie flat) or a contraindication to gadolinium CA (estimated Glomerular Filtration Rate <30 ml/min).

### Patients

32 patients with a history of stable angina, known or suspected CAD and a clinical indication for a diagnostic X-ray coronary angiography were recruited prospectively. These patients underwent a CMR examination including stress and rest perfusion prior to their angiogram. Exclusion criteria were the same as for volunteers and also an acute coronary syndrome within 6 weeks, and contraindication to adenosine (asthma, high grade atrioventricular node block).

### Data acquisition

All imaging was performed on a 1.5 T MR scanner (Achieva, Philips, Best, The Netherlands) and a 32-channel phased array receiver coil. The imaging parameters for both perfusion sequences are shown in Table 1. For both sequences a standard cosine-square filter with identical settings was used to reduce ringing artefacts. Standard reconstruction methods provided by the scanner software were used.

**Table 1:** **Imaging parameters used with the standard (st-GrE) and high-resolution (kt**_**BLAST**_**-SSFP) sequences**

**Parameter**	**st-GrE**	kt_BLAST_-SSFP
**Acquired spatial resolution**	2.6 × 2.8 × 10 mm	1.7 × 1.9 × 10 mm
**Echo time (TE)**	shortest (range 1.61–1.91 ms)	shortest (range 1.29–1.59 ms)
**Repetition time (TR)**	shortest (range 3.6–3.9 ms);	shortest (range 2.59–3.18 ms)
**Flip angle**	18°	50°
**Prepulse**	90°	90°
Prepulse delay*	100 ms	100 ms
**Acceleration technique**	SENSE: factor 2	kt-BLAST factor 5 with 11 training profiles (effective k-t factor 3.8).
**Image acquisition time**	140–152 ms	80–99 ms
**Water-fat shift**	0.438pixel	0.165pixel
**Bandwidth**	496 Hz	1316 Hz

*Prepulse delay: time between saturation preparation pulse and centre of k-space acquisition.

Volunteers underwent rest perfusion imaging using both st-GrE and kt_BLAST_-SSFP in addition to the clinical scan protocol. The order of the two perfusion sequences was alternated to control for the effect of higher baseline myocardial signal following the first administration of CA. There were no deviations from the perfusion sequence parameters listed in Table 1.

Patients were allocated sequentially to undergo either st-GrE or kt_BLAST_-SSFP perfusion imaging with the first 16 recruited to the st-GrE group and the second 16 to the kt_BLAST_-SSFP group. Patients underwent stress and rest perfusion, functional and scar imaging. Stress imaging always preceded rest imaging. In order to account for higher heart rates at stress, if required, the voxel size was increased stepwise, to maintain imaging at every heartbeat. This was required in 3 patients in the st-GrE group (resulting in a spatial resolution of 3.0 x 3.0 in 2 patients and 3.5 x 3.5 in 1 patient). Conversely voxel size did not need to be increased in any patients in the kt_BLAST_-SSFP group.

### Perfusion protocol

Patients were asked to abstain from smoking, caffeine and other adenosine antagonists for 24 h prior to imaging. Those who reported not to have followed this instruction were excluded (this restriction applied to patients only and not volunteers). Prior to entering the scanner room we emphasized the importance of following the breath-holding commands during perfusion imaging to all participants. A survey and coil sensitivity data were acquired at the beginning of the scan. Perfusion imaging was planned from the systolic phase of the 4 and 2-chamber cines. Three equally spaced short axis slices at basal, mid and apical left ventricular levels were acquired every heartbeat [14]. Ten-second test scans without CA using both sequences were performed first and any problems identified were addressed (e.g. artefacts, breath-holding or ECG problems). The imaging geometry and field of view were subsequently kept constant for both first pass perfusion scans.

All perfusion imaging was performed using a dual bolus of weight-adjusted gadolinium CA (Gadobutrol/Gadovist, Schering, Germany) in keeping with standard local perfusion imaging protocols as previously described [15]. The prebolus and main bolus consisted of 0.01 mmol/kg and 0.1 mmol/kg doses of CA respectively. Both doses of CA were flushed with 20 ml normal saline. All injections were performed by a power injector (Spectris Solaris® EP, MEDRAD, INC., USA). There was a 25 s delay between the prebolus and main bolus of CA.

All perfusion sequences were 70 s in duration (regardless of the number of heartbeats acquired). During perfusion image acquisition the patient was instructed to breath gently until delivery of the main bolus of contrast agent commenced at which point the patient was instructed to take a small breath in and hold for as long as possible. A breath hold of approximately 20 s was thus required to image the first pass of the main bolus of CA. For stress (patients only) an intravenous infusion of 140mcg/kg/min adenosine was administered for 4 min and 10 s. Imaging was commenced 3 min into the infusion.

### X-ray coronary angiography

X-ray coronary angiography was performed according to the standard Judkin’s technique. Multiple projections of the coronary arteries were acquired including at least two orthogonal views to assess stenosis severity.

### Data analysis

Data analysis was performed with the dedicated software CMR42 (Circle, Calgary, Canada) except where specifically stated otherwise.

### Image quality

Image quality was assessed qualitatively in all participants and graded on a 5-point scale (4 = excellent, 3 good, 2 moderate, 1 poor and 0 non diagnostic) by an expert observer, from within the department, who was blinded to the clinical details and the perfusion sequence used.

### Signal-to-noise and contrast-to-noise ratio

SNR and CNR were calculated from the mid ventricular slice in all 16 volunteers. Epi and endocardial borders were manually traced on the first image where the main bolus of CA was visible in the myocardium. The superior right ventricular insertion point was manually defined. This subsequently allowed 6 standard myocardial segments [16] to be automatically defined in each dynamic of the perfusion scan. This segmentation was manually corrected if required. Time versus signal intensity (SI) curves were then generated for each segment. Subsequently, the mean SI and the standard deviation (SD) of the SI in each segment on the frame with peak myocardial enhancement were measured. Baseline segmental mean SI and SD measurements were obtained for both sequences prior to any contrast injection. From these measurements, the segmental baseline and peak SNR of the myocardium were calculated as follows [9]:

(1) $$SNR_{\mathrm{baseline}}=Mean\;SI_{\mathrm{baseline}}/SD_{\mathrm{baseline}}$$

(2) $$SNR_{\mathrm{peak}}=Mean\;SI_{\mathrm{peak}}/SD_{\mathrm{baseline}}$$

Then, CNR values were calculated as follows:

(3) $$CNR=SNR_{\mathrm{peak}}-SNR_{\mathrm{baseline}}$$

### Image artefacts

The severity of respiratory artefacts was evaluated by the same blinded expert and graded as follows: 4 = nil significant, 3 = minor, 2 moderate 1 = severe, 0 = non-diagnostic due to respiratory artefact. Respiratory artefacts were defined as any artefact due to respiratory motion that impaired visualisation of the myocardium during the first pass of CA.

The extent, transmurality and duration of DRA were evaluated by a second, unblinded expert observer. The extent of DRA was defined as the percentage of segments affected. Transmurality was graded as 1 (1–25%), 2 (26-50%), 3(51–75%) or 4 (76–100%). Duration was recorded as the number of frames (and therefore heartbeats) for which the artefact was present.

### Diagnosis of CAD

Patient CMR studies and X-ray angiograms were evaluated by independent experts blinded to the results of the other investigation. CMR studies were classified as positive or negative for CAD. Angiograms were analysed visually and classified as positive or negative for the presence of at least one stenosis of ≥70% in a coronary artery ≥2 mm in diameter to allow calculation of a sensitivity and specificity for the detection of CAD.

### Statistical analysis

Statistical analysis was performed using IBM SPSS Statistics version 19. Data are expressed as the mean ± SD. Differences between the two groups of patients were compared using unpaired t tests or Fisher’s exact test for normally distributed or non-parametric data respectively. Image quality and respiratory artefacts scores were compared using the Wilcoxon signed ranks test or the two-sample Kolmogorov-Smirnov test in volunteers and patients respectively. Mean scores for SNR, CNR and DRA were compared using paired t tests for within group and unpaired t-tests for between group comparisons. Significance was determined at <0.05.

## Results

### Study population

All volunteer studies were completed successfully. The CMR examination was normal in 13 volunteers. Three volunteers were found to have slightly abnormal scans: 2 had mildly impaired left ventricular function whilst 1 had a mildly dilated right ventricle. These were isolated abnormalities in each case.

One patient from the st-GrE group did not complete the protocol due to claustrophobia and was excluded. Participant characteristics are shown in Table 2. Patients in both the st-GrE and kt_BLAST_-SSFP arms were well matched for age, sex, body mass index and cardiovascular risk factors. There were significantly more patients who had undergone previous percutaneous coronary intervention (PCI) in the kt_BLAST_-SSFP arm and these patients also had a lower systolic blood pressure at rest. However, heart rate, systolic blood pressure during stress and rate pressure product were not different.

**Table 2:** Study participant characteristics

	**Volunteers**	**Patients**		
		**st-GrE**	kt_BLAST_-SSFP	**p value**
**Age**	43 ± 20	64 ± 9	64 ± 11	0.89
**Male**	8 (50%)	10 (67%)	13 (81%)	0.3
**Body Mass Index (kg/m**^**2**^**)**	24 ±4	30 ± 5	28 ± 4	0.21
**Diabetes**	0	6 (40%)	7 (44%)	1
**Hypertension**	0	12 (80)	9 (56%)	0.25
**Smoker**	0	2 (13%)	2 (13%)	1
**Previous PCI**	0	0	4 (25%)	0.02
**LVEF**	58 ± 7%	58 ±12%	63 ±10%	0.23
**RVEF**		59 ± 8%	60 ± 8%	0.67
**CAD**	0	12 (80%)	13 (81%)	1
**Scar present**	0	5 (33%)	2 (13%)	0.22
**Haemodynamics**				
**HR rest**	69 ± 10	64 ± 15	65 ± 11	0.8
** stress**		85 ± 20	84 ± 15	0.93
**SBP rest**		148 ± 21	132 ± 17	0.03
** stress**		142 ± 21	129 ± 23	0.15
**RPP rest**		8851 ± 2545	8562 ± 1950	0.74
** stress**		10942 ± 2319	10823 ± 2662	0.91

p values refer to differences between patients in the st-GrE and the kt_BLAST_-SSFP group.

PCI = percutaneous coronary intervention, LVEF: left ventricular ejection fraction; RVEF: right ventricular ejection fraction; HR: heart rate (beat per minute); SBP: systolic blood pressure (mmHg); RPP: rate-pressure product \(HR × SBP).

### Image quality

#### Qualitative assessment

**Figure 1:**
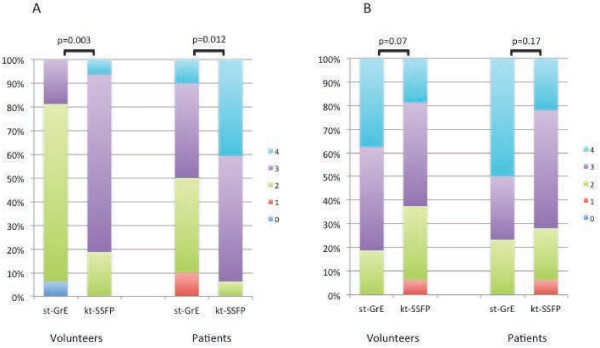
**Image quality and respiratory artefact scores for volunteers and patients with both sequences.** Scores for image quality **(A)** and respiratory artefacts **(B)** in volunteers and patients ranging from 0 to 4 (4 = excellent, 3 good, 2 moderate, 1 poor and 0 non diagnostic for image quality; 4 = nil significant, 3 = minor, 2 moderate 1 = severe, 0 = non-diagnostic due to respiratory artefact for respiratory artefacts). Image quality scores were significantly higher with kt_BLAST_-SSFP and there was a non-significant trend towards fewer respiratory artefacts with st-GrE.

Image quality was significantly better with kt_BLAST_-SSFP compared to st-GrE in both volunteers (p = 0.003) and in patients (p = 0.012) (Figure 1). Image quality was good or excellent in 19% of volunteers with st-GrE compared to 81% with kt_BLAST_-SSFP. In patients 50% of images were good or excellent with st-GrE whereas the corresponding figure for kt_BLAST_-SSFPwas 94%.

#### SNR and CNR

**Figure 2:**
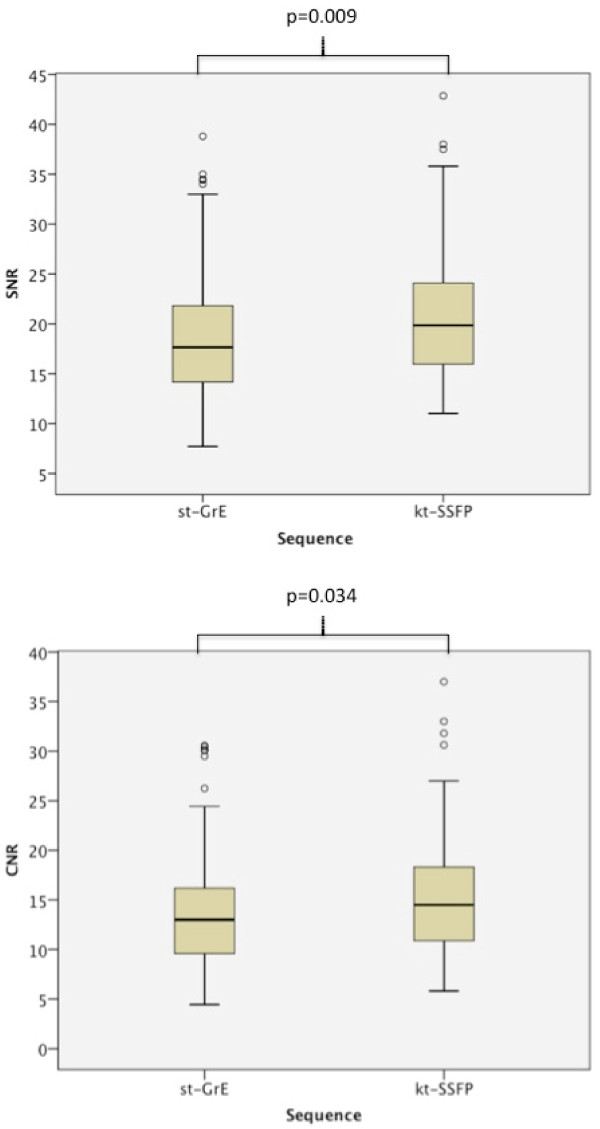
**Signal and contrast to noise ratios for volunteer rest perfusion scans.** Boxplots showing segmental signal to noise (SNR) and contrast to noise (CNR) values in the volunteers with each sequence. Both SNR and CNR were significantly higher with kt_BLAST_-SSFP.

**Figure 3:**
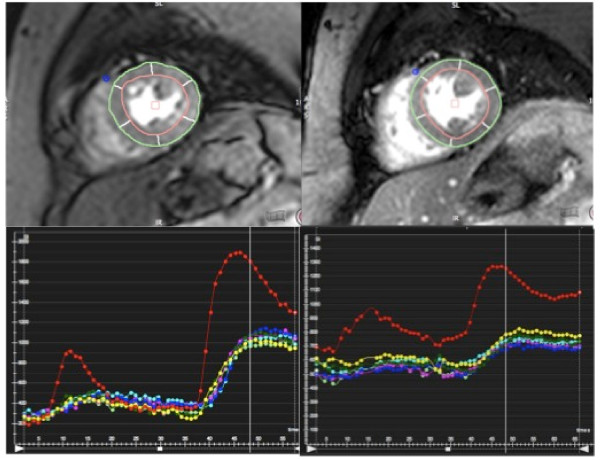
**First pass rest perfusion images with segmental signal intensity curves.** Segmented mid-ventricular slice during first-pass rest-perfusion with the st-GrE (left) and kt_BLAST_-SSFP sequences (right) with corresponding signal intensity curves. A dual bolus of contrast is used as standard but full-quantification was not performed as part of this study. The red curve is the left ventricular blood pool signal. All other colours represent the signal from each of the six standard segments. Baseline signal is lower with the st-GrE sequence as this sequence was used first in this volunteer.

SNR and CNR were both significantly higher with the kt_BLAST_-SSFP sequence (Figure 2). SNR was 18.8 ± 6.6 with st-GrE and 21.0 ± 6.7 with kt_BLAST_-SSFP (p = 0.009). CNR was 14.0 ± 6.0 with st-GrE and 15.4 ± 6.1 with kt_BLAST_-SSFP (p = 0.034). Myocardial signal intensity was lower with kt_BLAST_-SSFP compared to st-GrE but noise was reduced proportionately more resulting in increased SNR. Segmental signal intensity curves from a volunteer are shown in Figure 3.

### Image artefacts

#### Respiratory artefacts

There was a non-significant trend for increased respiratory artefacts with the kt_BLAST_-SSFP sequence in both volunteers (p = 0.07) and patients (p = 0.17) (Figure 1). No studies were non-diagnostic due to respiratory artefact. No st-GrE studies had severe respiratory artefacts in either patients or volunteers whereas with the kt_BLAST_-SSFP sequence severe respiratory artefacts affected both rest and stress scans in one patient and also one volunteer scan. In volunteers 38% of st-GrE scans compared to 19% of kt_BLAST_-SSFP were free of any respiratory artefact. In patients the corresponding figures were 50% and 22% respectively. Respiratory artefacts consisted of ghosting in space and time due to rapid motion caused by respiration. Movies demonstrating typical respiratory artefacts from both sequences are shown in Additional file 1 and 2.

#### Dark rim artefacts

The extent, and transmurality of DRAs was significantly lower with kt_BLAST_-SSFP in both volunteers and patients. The duration of DRA was also significantly less in patients with kt_BLAST_-SSFP although there was no difference with volunteers. These findings are summarised in Table 3. DRA was apparent in 39% of segments in volunteers and 33% in patients with st-GrE compared to 15% and 12% with kt_BLAST_-SSFP. DRA involved the basal slice most frequently (64–76% of affected segments), followed by the mid ventricular segments (24–33%), and the apical segments least often (0–5%).

**Table 3:** Dark rim artefacts

	***Volunteers***			***Patients***		
**Dark rim artefact**	**st-GrE**	kt_BLAST_-SSFP	**p value**	**st-GrE**	kt_BLAST_-SSFP	**p value**
Extent (%)	39 ± 13	15 ± 12	<0.0001	33 ± 14	12 ± 10	<0.0001
Transmurality	1.83 ± 0.58	1.02 ± 0.06	<0.0001	1.57 ± 0.40	1.07 ± 0.17	<0.0001
Duration	10.6 ± 3.56	9.7 ± 2.51	0.373	10.8 ± 3.7	8.0 ± 2.7	0.004

Extent refers to the percentage of segments affected.

Transmurality was scored as 1 = 1–25%, 2 = 26–50%, 3 = 51–75% and 4 = 76–100%. Duration refers to the number of frames for which the defect was visible.

The p values relate to the difference between the scores with st-GrE and kt_BLAST_-SSFP in both patients and volunteers.

#### Diagnosis of CAD

**Figure 4:**
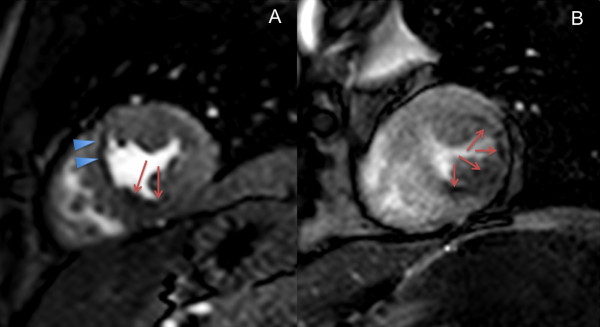
**Stress perfusion images from patients with coronary artery disease using both sequences.** Still images from the first pass of contrast agent during adenosine stress from two different patients. 4a is a st-GrE image in a patient with angina. There is a perfusion defect in the inferior/inferoseptal wall (arrows). There is also dark rim artefact visible, particularly in the anteroseptal segment (arrowheads). The patient was subsequently found to have an occluded right coronary artery. 4b is from another patient with angina. The lateral wall is thinned whilst the inferior/inferolateral wall is of normal thickness. There is a subendocardial perfusion defect in both of these regions (arrows). Late gadolinium enhancement revealed scar in the lateral wall but not the inferior/inferoseptal wall (not shown). This patient had a severe lesion in his proximal right coronary artery and an occluded circumflex artery.

Sensitivity and specificity for the detection of CAD against X-ray angiography were not significantly different: st-GrE 82% (48–97%) and 100% (31–100%), kt_BLAST_-SSFP 78% (40–96%) and 80% (30–99%) respectively. An example of a perfusion defect in one patient from each group is shown in Figure 4.

## Discussion

This study demonstrates that newer, advanced imaging techniques can improve the resolution of perfusion imaging and also result in significantly improved image quality, SNR and CNR and significantly reduced DRA.

CMR has emerged relatively recently as a valuable tool for the assessment of cardiac patients. However on-going technical developments have resulted in continuing rapid evolution of CMR and perfusion techniques. It is difficult to predict the effects of sequence alterations on image quality and artefacts or diagnostic accuracy and as such it is necessary to continually evaluate new methods against those that have been previously established through clinical trials and practice. We therefore sought to compare the performance of a state-of-the art perfusion sequence with an optimised established sequence. We evaluated both sequences in normal hearts and in patients. This provided a comprehensive and clinically relevant assessment of image quality and artefacts, and also SNR and CNR.

A number of different types of CMR sequence can be used for perfusion imaging and we have only compared two in this study. However, each sequence also has very many parameters, each of which can be adjusted to influence imaging. Consequently, a large number of permutations and combinations exist which cannot all realistically be compared. We therefore opted to comprehensively compare an optimised standard perfusion sequence to a state-of-the-art sequence. An ultrafast gradient echo sequence was chosen as the standard sequence as it is very commonly used in the clinical setting and has been used in important recent perfusion CMR studies [17]. Our advanced sequence was not simply a modification of this but a sequence that we felt was most likely to produce results of the highest quality using technology that is widely available.

Previous studies have evaluated *k-t* acceleration techniques for improving perfusion imaging. In keeping with our findings a study by Maredia et al. in 10 normal volunteers found reduced DRA with a *k-t* SENSE accelerated gradient echo sequence compared to a reference SENSE accelerated sequence [18]. These authors used *k-t* acceleration to improve spatial resolution, temporal resolution or a combination of both and found that maximizing spatial resolution produced the greatest reduction in DRA.

Another study compared *k-t* SENSE accelerated gradient echo sequences in 14 volunteers and in 37 patients at 1.5 and 3 T [19]. In addition a standard lower resolution SENSE accelerated gradient echo sequence was compared to a *k-t* accelerated sequence at 3 T. This study also demonstrated improved image quality and reduced DRA with the *k-t* accelerated sequence compared to the standard sequence. In keeping with our study they did not demonstrate improved diagnostic accuracy for CAD with the higher resolution sequence in this relatively small study. Our current study builds on these previous studies as we have demonstrated consistent findings using a *k-t* BLAST accelerated balanced sequence, in both patients and volunteers, at 1.5 T. At present 1.5 T scanners are used most commonly for clinical perfusion imaging despite the potential advantages associated with imaging at 3 T.

In this study the use of *k-t* acceleration allowed higher resolution imaging with preservation of three-slice coverage of the heart each heartbeat even at higher heart rates. Higher spatial resolution may improve the detection of sub-endocardial perfusion defects and thus result in improved sensitivity for CAD. To date this has not been confirmed in clinical studies, however, limited early data support the possibility that high-resolution techniques may be more accurate [20]. Furthermore, in patients with higher heart rates during stress perfusion imaging, the constraints of the sequence may make it necessary to reduce spatial resolution, cardiac coverage or temporal resolution by imaging on alternate heartbeats. This occurred in 3 patients in the st-GrE group and spatial resolution had to be reduced further but higher speed-up with *k-t* acceleration meant that it did not occur in the kt_BLAST_-SSFP group despite the higher spatial resolution of this sequence. Lower temporal resolution may also compromise diagnostic accuracy and, for example, is known to affect the calculation of a myocardial perfusion reserve index [21].

### Image quality

Although image quality was at least moderate in almost all volunteers and patients with both sequences kt_BLAST_-SSFP images were significantly better and most were scored as good or excellent. Perfusion image quality is important, as good quality high-resolution scans are required to accurately delineate regions of ischaemia. This is particularly required for evaluating epi-endocardial differences in perfusion or, in combination with late gadolinium enhancement imaging, for establishing whether there is peri-infarct ischaemia. Such findings can be useful diagnostically and also for patient management, for example when decisions are made regarding revascularization. This study was too small to demonstrate whether improved image quality results in a difference in diagnostic performance. However, previous work has demonstrated that sensitivity to perfusion defects and inter-observer reproducibility are related to image quality and also to SNR [22].

### SNR and CNR

The kt_BLAST_-SSFP sequence voxel size was approximately half that of the st-GrE sequence and consequently a reduction in SNR and CNR by a factor of 2 may have been expected. However, our data show that this is not the case. The use of a balanced sequence, which results in higher signal has compensated for the reduction in voxel size. Previous studies [10,23] have also found a higher SNR and CNR with a balanced SSFP sequence compared to a GRE perfusion sequence.

Noise can vary greatly across the field of view with the use of parallel imaging [24] and thus SNR and CNR measurements can be difficult. We measured signal and noise from standard myocardial segments defined on the reconstructed images and demonstrated an improved SNR and CNR with the kt_BLAST_-SSFP sequence. Whilst the SNR and CNR values obtained from the reconstructed images may not be the same as the true values they remain relevant as the reconstructed images are the ones used for clinical interpretation. SNR and CNR were not measured in patients given the heterogeneous nature of pathologic perfusion defects. We used the mid-ventricular slice to minimise partial volume effects which are more likely to occur in the basal and apical slices and also DRA which occurred more frequently in the basal slice. In addition the use of automatically-generated myocardial segments as regions of interest resulted in a standardised, less user-dependent, approach.

### Artefacts

DRA is a well-known and important weakness of perfusion imaging. A dark rim appears with the arrival of the CA bolus in the left ventricle and obscures the border of the endocardium and the left ventricular blood pool. DRA is related to juxtaposition of the high signal from blood pool and low signal from myocardium and is often reported as transient. However if the blood pool remains bright, for example if the arrival of the bolus is dispersed, it can be persistent. DRA can therefore be particularly troublesome given that pathological perfusion also results in dark areas of myocardium and can make image interpretation difficult even for experienced observers. Furthermore quantitative analysis of perfusion relies on obtaining accurate myocardial SI curves and can also be severely compromised by the presence of DRA. Inclusion of DRA within regions of interest will result in incorrect perfusion values, particularly from the sub-endocardium. Myocardial borders can be manually defined to exclude areas of DRA however automated algorithms for myocardial border detection are unlikely to be able to accurately differentiate DRA from true-defects. In turn quantitative assessment of perfusion are unlikely to make the transition from research tool to clinically useful tool without robust and rapid automated methods.

The causes of DRA are incompletely understood but cardiac motion during image acquisition and lower spatial resolution are suspected to contribute [25]. In this study DRA was significantly reduced with the kt_BLAST_-SSFP sequence. This may be a result of increased spatial resolution reducing truncation artefacts and/or the shorter acquisition time and reduced cardiac motion during acquisition. However, it would have been difficult to predict this in advance, as the increased difference in signal between the blood pool and myocardium expected with a balanced SSFP sequence could have resulted in increased DRA.

Respiratory artefacts can also compromise qualitative and quantitative perfusion imaging particularly stress imaging in patients with cardiac pathology when breath holding in combination with adenosine stimulation can be challenging. Temporal under-sampling with the kt_BLAST_-SSFP sequence would be expected to exacerbate this problem. Respiratory artefacts were seen as ghosting in space and time and were worst with large respiratory movements. Although correct breath holding resulted in fewest artefacts kt_BLAST_-SSFP associated respiratory artefacts were usually only minor when subjects continued to take shallow breaths and in general were only slightly more severe than those we encountered during non-*k-t* perfusion imaging. However, although there was a trend towards more respiratory artefact with kt_BLAST_-SSFP it was not significant. The phase encoding direction with both sequences in this study was antero-posterior however it may be possible to further reduce kt_BLAST_-SSFP sequence respiratory artefacts by using a the head-foot direction, although this may itself subsequently increase acquisition times.

We did not encounter any other significant artefacts, such as ECG mistriggering, with either sequence in this study. Methodical patient preparation and attention to the test scan allows prevention or correction of the majority of such problems prior to the perfusion imaging.

### Limitations

It may have been preferable to use both sequences in the same patients rather than two different groups. However our patients were well matched overall. Both sequences were compared in the same conditions in the same volunteers and the quantitative assessments were performed in these groups. Performing perfusion imaging with both sequences in patients also has problems; either stress perfusion has to be repeated on the same day (possibly at the expense of rest perfusion due to CA dose limitations) or patients have to attend on two occasions, when conditions such as myocardial perfusion or patient positioning in the magnet, may not be the same.

The half-life of the CA used is approximately 90 min (depending on renal function) [26]. Therefore after the first dose of CA, myocardial signal does not reduce to baseline within the timeframe of a single CMR examination (as seen in Figure 3). To account for this we alternated which perfusion sequence we used first in volunteers. Inadequate coil response non-uniformity correction may also have resulted in variations in signal intensity the field of view in some cases. However since the same segments were compared with both sequences this would not have affected the overall result.

Finally, a relatively small number of patients were included in the study, which means that it was not possible to determine whether there is a difference in diagnostic performance between the two sequences. However each sequence was comprehensively assessed in volunteers and in patients. This study therefore provides a platform for larger scale studies to evaluate whether CMR sequences using advanced techniques also improve diagnostic accuracy.

## Conclusion

Advanced, high-resolution perfusion CMR using a *k-t* accelerated SSFP technique results in significantly improved image quality, signal and contrast to noise ratios and a reduction in dark-rim artefacts. These findings support the use of an advanced high-resolution sequence in preference to a standard sequence for clinical myocardial perfusion imaging. However further studies exploring whether the use of advanced methods can be translated into superior diagnostic accuracy for coronary disease are desirable.

## Competing interests

Eike Nagel has received grant support from Philips Healthcare and Bayer Schering Pharma. The other authors declare that they have no competing interests.

## Authors contributions

GM designed the study protocol, acquired and analysed the data and drafted the manuscript. MI, AS, SH and AC helped acquire and analyse the data and critically revised the manuscript, EN and TS assisted with study design and interpretation of data and critically revised the manuscript. All authors read and approved the final manuscript.

## Supplementary Material

Additional file 1 **Movie 1.** Rest perfusion from a patient demonstrating a typical respiratory artefact seen with st-GRE sequence. Motion due to respiration results in ghosting due to coil-profile data misregistration from the anterior coil. This is seen best at the beginning of the first pass of contrast agent where contrast from the right ventricle appears to be within the septum. This artefact was classified as mild. This movie also demonstrates dark rim artefact typical of this sequence. Click here for file

Additional file 2 **Movie 2.** Stress perfusion from a patient demonstrating a typical respiratory artefact seen with st-GRE sequence. The participant’s breath hold is late and short and respiratory motion again results in ghosting in time and space best seen at the beginning and towards the end of the movie. However as the myocardium is well visualised for the majority of the first pass despite the artefact, and a perfusion defect clearly visualised in the infero-lateral region, this was classified as a moderate artefact. Click here for file
